# Chronobiology in Paediatric Neurological and Neuropsychiatric Disorders: Harmonizing Care with Biological Clocks

**DOI:** 10.3390/jcm13247737

**Published:** 2024-12-18

**Authors:** Gabriele Giannotta, Marta Ruggiero, Antonio Trabacca

**Affiliations:** 1 Associazione “La Nostra Famiglia”, IRCCS “E. Medea”, Scientific Hospital for Neurorehabilitation, Unit for Severe Disabilities in Developmental Age and Young Adults, Developmental Neurology and Neurorehabilitation, 72100 Brindisi, Italy; gabriele.giannotta@lanostrafamiglia.it (G.G.); marta.ruggiero@lanostrafamiglia.it (M.R.); 2 Scientific Institute IRCCS “E. Medea”, Scientific Direction, 23842 Bosisio Parini, Italy

**Keywords:** chronobiology, circadian rhythms, paediatric neurology, neuropsychiatric disorders, chronotherapy

## Abstract

**Background**: Chronobiology has gained attention in the context of paediatric neurological and neuropsychiatric disorders, including migraine, epilepsy, autism spectrum disorder (ASD), attention-deficit/hyperactivity disorder (ADHD), and post-traumatic stress disorder (PTSD). Disruptions in circadian rhythms are associated with key symptoms such as sleep disturbances, mood dysregulation, and cognitive impairments, suggesting a potential for chronobiology-based therapeutic approaches. **Methods**: This narrative review employs a systematic approach to identify relevant studies through searches of three major scientific databases, NCBI/PubMed, ScienceDirect, and Scopus, up to July 2024. We used a combination of broad and condition-specific keywords, such as “chronobiology”, “biorhythm”, “pediatric”, “epilepsy”, “ADHD”, and “ASD”, among others. Articles in English that focused on clinical features, treatments, or outcomes related to circadian rhythms in paediatric populations were included, while non-peer-reviewed articles and studies lacking original data were excluded. Rayyan software was used for article screening, removing duplicates, and facilitating consensus among independent reviewers. **Results**: A total of 87 studies were included in the analysis. Findings reveal a consistent pattern of circadian rhythm disruptions across the disorders examined. Specifically, dysregulation of melatonin and cortisol secretion is observed in children with ASD, ADHD, and PTSD, with altered circadian timing contributing to sleep disturbances and mood swings. Alterations in core clock genes (*CLOCK*, *BMAL1*, *PER*, and *CRY*) were also noted in children with epilepsy, which was linked to seizure frequency and timing. Chronotherapy approaches showed promise in managing these disruptions: melatonin supplementation improved sleep quality and reduced ADHD symptoms in some children, while light therapy proved effective in stabilizing sleep–wake cycles in ASD and ADHD patients. Additionally, behaviour-based interventions, such as the Early Start Denver Model, showed success in improving circadian alignment in children with ASD. **Conclusions**: This review highlights the significant role of circadian rhythm disruptions in paediatric neurological and neuropsychiatric disorders, with direct implications for treatment. Chronobiology-based interventions, such as melatonin therapy, light exposure, and individualized behavioural therapies, offer potential for improving symptomatology and overall functioning. The integration of chronotherapy into clinical practice could provide a paradigm shift from symptom management to more targeted, rhythm-based treatments. Future research should focus on understanding the molecular mechanisms behind circadian disruptions in these disorders and exploring personalized chronotherapeutic approaches tailored to individual circadian patterns.

## 1. Introduction

Biorhythms play an essential role in regulating various physiological processes in the human body [1]. These rhythms—circadian (approximately 24 h cycles), infradian (cycles longer than 24 h), and ultradian (cycles shorter than 24 h)—govern key functions such as the sleep–wake cycle, hormone release, and core body temperature [2]. Among these, circadian rhythms, driven by the suprachiasmatic nucleus (SCN) of the hypothalamus, are the most studied and well understood [3]. The SCN acts as the body’s master clock, synchronizing internal physiological processes with external cues, primarily light and dark [4].

In recent decades, there has been growing recognition of how disruptions in circadian rhythms can negatively influence or even trigger various health conditions [5]. The molecular clock’s components are broadly expressed throughout the brain, and the genes’ ability to alternate between transcriptional activation and repression suggests they may orchestrate circadian control over neuronal gene expression and activity. This orchestration influences the function of neurotransmitters and receptors involved in the regulation of emotion and cognition [5]. For example, projections from the SCN to the locus coeruleus (LC) facilitate circadian regulation of noradrenergic activity and are important for transitions from focused attention to behavioural flexibility [6], contextual fear conditioning, circadian regulation of the sleep–wake cycle [7], and synaptic plasticity, including long-term potentiation [8]. Chronobiology, the scientific study of biological rhythms [9], delves into the profound interplay between these rhythms and human health. Research reveals that biological rhythms play a pivotal role in the pathophysiology of several diseases, ranging from metabolic disorders to cardiovascular conditions [10,11]. Neurological conditions, particularly in paediatric populations, have emerged as key areas where biological rhythms may significantly impact disease progression and symptom manifestation [12]. Abnormalities in sleep–wake rhythms, appetite, and social rhythms have been also observed in depressive disorders, schizophrenia, bipolar disorder, anxiety disorders, seasonal affective disorder (SAD), and a variety of other CNS disorders [13,14]. Recent studies have showed that disruption of one of the core proteins in the master circadian clock can trigger mania-like behaviours [15], and human genetic studies have associated polymorphic variations of the clock and clock-related genes with mood disorders [16,17], SAD [18], and autism spectrum disorders (ASD) [19,20], suggesting the involvement of circadian genes in these disorders. Owing to the developing brain’s increased vulnerability to environmental and internal rhythm disturbances, recognizing the cyclic nature of symptoms in paediatric neurological conditions could provide innovative pathways for therapeutic advancement. The developing brain is especially sensitive to disruptions in circadian timing, which may exacerbate symptoms or alter disease trajectories in children [12]. Furthermore, conditions such as epilepsy, migraine, autism spectrum disorder (ASD), attention-deficit/hyperactivity disorder (ADHD), and post-traumatic stress disorder (PTSD) have been shown to exhibit time-of-day-dependent symptom patterns intricately linked to circadian rhythm disturbances [20,21,22,23,24].

In paediatric populations, these disruptions may lead to irregularities in the timing and severity of symptoms, complicating diagnosis and treatment. For instance, children with epilepsy often experience seizures that follow circadian patterns, while those with ADHD may show fluctuations in attention and hyperactivity aligning with disruptions to their biological clocks [25]. Similarly, sleep disturbances in ASD and PTSD may reflect deeper circadian misalignments, contributing to symptom worsening and increased vulnerability to comorbid conditions [26].

Despite substantial evidence linking circadian disruptions to these disorders, much of the current research remains predominantly correlational [27], and the integration of chronobiological principles into clinical practice remains limited. This narrative review aims to bridge this gap by synthesizing existing research to identify circadian patterns in paediatric neurological and neuropsychiatric conditions. By examining the implications of circadian rhythms on symptomatology and treatment, we aim to provide a comprehensive overview of how chronobiology could inform novel, circadian-based therapeutic interventions.

The novelty of this review lies in its emphasis on moving beyond symptom management toward chronotherapy-based interventions, which align treatment with the patient’s natural rhythms. By advancing our understanding of circadian contributions to these disorders, we highlight the potential for chronobiology to reshape treatment strategies for children with neurological and neuropsychiatric conditions.

## 2. Methods for the Literature Search

This study was structured as a narrative clinical review, applying a systematic approach to the literature search and article selection [28]. By integrating systematic methods within a narrative framework, we aimed to comprehensively synthesize existing research on the influence of circadian rhythms on paediatric neurological and neuropsychiatric conditions [29].

The literature search targeted three primary scientific databases: NCBI/PubMed, ScienceDirect, and Scopus, covering publications available up to June 2024. The search terms combined broad and specific keywords related to paediatric neurological and neuropsychiatric conditions influenced by chronobiology. Keywords included terms like “chronobiology”, “biorhythm”, “pediatric”, “migraine”, “headache”, “epilepsy”, “autism spectrum disorder (ASD)”, “attention-deficit/hyperactivity disorder (ADHD)”, and “post-traumatic stress disorder (PTSD)”. Boolean operators were employed to enhance search specificity, and search strategies were adjusted to meet each database’s syntax requirements. Inclusion criteria specified articles written in English that examined clinical features, symptoms, therapeutic approaches, or outcomes related to the impact of circadian rhythms on paediatric neurodevelopmental and neuropsychiatric conditions. Exclusion criteria encompassed conference abstracts, editorials, news articles, opinion pieces, discussion papers, and studies focusing exclusively on adult populations. Non-peer-reviewed articles and papers without original data were excluded. To ensure comprehensive coverage, additional relevant articles were identified through manual review of references in key studies, though grey literature and unpublished works were not considered.

To manage the article selection process, we utilized Rayyan, a web-based tool designed for systematic reviews [30]. Rayyan facilitates independent screening of titles and abstracts, removes duplicates automatically, and reduces bias through its “blind” function. Its text-mining and labelling features enhance workflow efficiency, enabling simultaneous abstract screening and full-text retrieval, ensuring a streamlined review process [27].

The initial database search results were screened by title and abstract, with Rayyan automatically removing duplicates. Two independent reviewers conducted a full-text review of eligible studies to verify alignment with the inclusion criteria. For any disagreements, Rayyan’s “blind” function facilitated independent evaluations, followed by discussions to achieve consensus. A third reviewer was consulted as needed to resolve any remaining discrepancies. Only articles meeting all inclusion criteria were included in the final analysis.

Ultimately, 87 articles met the inclusion criteria, focusing on the clinical features, symptomatology, therapeutic interventions, and circadian impacts on paediatric neurodevelopmental and neuropsychiatric conditions. The selected literature was critically analysed for relevance to our review on the central role of chronobiology and chronotherapy in paediatric neurology and psychiatry.

## 3. Chronobiology and Neurological Conditions

### 3.1. Migraine

Headaches, particularly migraines and tension-type headaches, are among the most common neurological complaints in paediatric patients [31,32]. Emerging evidence underscores the significant influence of circadian rhythms on these conditions [21]. Migraines, in particular, exhibit a pronounced circadian pattern, often peaking in the early morning and late afternoon [33]. This temporal occurrence is associated with fluctuations in key neurohormones, including cortisol, melatonin, and serotonin, all of which follow circadian cycles [34]. Notably, reduced evening melatonin levels are frequently observed in paediatric migraine sufferers, suggesting that circadian misalignment may contribute to migraine onset [35].

The hypothalamus, specifically the suprachiasmatic nucleus (SCN), has also been implicated in the pathogenesis of migraines [36,37]. Increased hypothalamic activity has been documented during the prodromal phase of migraine attacks, suggesting that dysfunction in this circadian-regulated region could trigger migraine episodes [37]. Disruptions in melatonin secretion, governed by the SCN and produced by the pineal gland, further exacerbate sleep disturbances in children with migraines, particularly those with comorbid conditions such as insomnia [38]. Moreover, research indicates a higher prevalence of evening chronotype (a preference for later sleep and wake times) among children with migraines, a sleep pattern linked to poor sleep quality and increased headache frequency [39].

Both sleep regulation and migraine mechanisms share common neurochemical and neuroanatomical pathways. Dysregulation of neurotransmitters like serotonin and dopamine, which are involved in both sleep-stage transitions and the early symptoms of migraines (e.g., yawning, mood changes), underscores the shared pathophysiology between these processes [37]. Circadian disruptions, including irregular sleep schedules and night-time light exposure, suppress melatonin production, worsening both sleep quality and migraine frequency [40].

Research also indicates a relationship between early childhood sleep disturbances, such as colic, and the subsequent development of migraines [41,42,43,44,45]. Several studies have shown that infantile colic, traditionally seen as a gastrointestinal issue, could actually reflect a disruption in sleep patterns, increasing the risk of migraines in later childhood [43]. One review found that infant colic is associated with an increased odds ratio of developing migraines, pointing to shared pathophysiological mechanisms, probably related to the regulation of circadian rhythms [46]. This evidence emphasizes the importance of addressing early sleep disturbances, such as infant colic, to potentially mitigate the risk of chronic headache disorders linked to biorhythm imbalances [47]. 

Sleep disturbances are frequently reported in children with migraines, including insomnia, parasomnias, and obstructive sleep apnoea (OSA) [44,45]. Studies indicate that parasomnias, such as sleepwalking and night terrors, affect up to 30% of paediatric migraine sufferers, a rate significantly higher than that observed in the general population [44,45]. Additionally, OSA (a well-established cause of morning headaches) is prevalent among children with migraines [48]. 

The complex interplay between circadian rhythms, sleep disorders, and paediatric migraines highlights the need for a comprehensive approach to diagnosis and treatment. The overlap in brain structures and neurochemical pathways involved in both sleep regulation and migraine pathophysiology suggests a shared biological foundation. By addressing circadian misalignment and improving sleep quality, clinicians can better manage paediatric migraines.

### 3.2. Epilepsy

Paediatric epilepsy is a neurological disorder characterized by recurrent, unprovoked seizures in children, often with multifactorial causes that range from genetic to structural abnormalities [49]. Its impact extends beyond seizures, affecting cognitive, behavioural, and sleep functions [50]. Understanding how biological rhythms influence epilepsy is crucial, especially in paediatric populations where brain development is ongoing. Circadian rhythms play a central role in modulating epileptic activity. While sleep disorders are commonly observed in children with epilepsy, the relationship between circadian rhythms and seizure patterns offers a further framework for understanding and managing the condition [22].

Seizures in paediatric epilepsy exhibit distinct temporal patterns, strongly influenced by the circadian cycle. These rhythms govern various neurophysiological processes, including cortical excitability, which in turn modulates the likelihood of seizure occurrence. For example, generalized seizures in children are more likely to occur in the early morning following sleep, while certain focal seizures, such as those arising from the frontal lobe, occur predominantly at night [51]. The underlying mechanism involves circadian genes like *BMAL1* and *CLOCK*, which regulate the excitability of neurons and thus influence seizure thresholds [52,53].

In paediatric cases, these circadian influences are even more pronounced because the developing brain is particularly sensitive to changes in the sleep–wake cycle [54]. Seizure activity tends to follow the natural fluctuations of cortical excitability that occur throughout the day, with specific epilepsy syndromes showing different peaks in seizure frequency [55]. 

The overlap between circadian rhythms and sleep disorders in paediatric epilepsy suggests that these conditions may share common regulatory pathways [22]. The disruption of circadian rhythms often leads to disturbances in sleep patterns, which can manifest as insomnia or hypersomnia in children with epilepsy [56]. These circadian misalignments not only predispose individuals to seizures but also impair the regulation of sleep–wake cycles, resulting in chronic sleep disturbances [57]. The circadian clock also interacts with the mTOR signalling pathway, which has been implicated in both epileptogenesis and sleep regulation [52,53]. Moreover, the type of epilepsy may also determine the severity and nature of sleep disruptions. For instance, children with sleep-related hypermotor epilepsy (SHE) often experience particularly pronounced disturbances in sleep architecture, including frequent nocturnal awakenings and abnormal motor activity during sleep [58]. The presence of comorbid sleep disorders, such as OSA or insomnia, further complicates epilepsy management and can lower quality of life [59]. 

A better understanding of how these two systems interact can lead to more effective therapeutic strategies. As research continues to unravel the connections between circadian biology and epilepsy, there is great potential for improving the management and quality of life for children living with this challenging condition.

## 4. Chronobiology and Neuropsychiatric Conditions 

### 4.1. Autism Spectrum Disorder

According to the World Health Organization, autism spectrum disorder (ASD) is a neurodevelopmental condition characterized by impairments in social interaction, communication, the presence of rigid or repetitive behaviours, and differences in the perception of sensory stimuli [60]. Over recent decades, increasing evidence has pointed toward a significant role of circadian rhythm disruptions in ASD, with wide-reaching effects on both the core symptoms of autism and the overall quality of life [20]. Chronobiology has become a growing area of interest in understanding ASD pathophysiology, focusing on the molecular mechanisms that underlie circadian dysregulation and its impact on cognitive, behavioural, and physiological processes [61]. The molecular foundation of circadian rhythms involves a set of core clock genes, including *CLOCK*, *BMAL1*, *PER1-3*, and *CRY1-2*, which participate in transcriptional–translational feedback loops (TTFLs) that generate rhythmic oscillations in gene expression and protein synthesis [62].

In ASD, dysregulation of circadian rhythms is a pervasive issue that is associated with both molecular and behavioural abnormalities. Melatonin and cortisol have been found to exhibit abnormal rhythmicity in individuals with ASD [63]. Melatonin is essential for regulating sleep–wake cycles and is typically secreted in response to darkness. Studies have consistently shown reduced nocturnal melatonin levels and a blunted circadian rhythm of melatonin secretion in individuals with ASD [64]. This reduction is not only correlated with sleep disturbances but also linked to broader behavioural symptoms, such as social interaction difficulties and heightened anxiety [65]. It is important to note that individuals with autism who also experience seizures often display an abnormal melatonin rhythm, which has been linked to electroencephalogram (EEG) changes [66]. Moreover, individuals with autism appear to be highly sensitive not only to disruptions in external environmental rhythms but also to internal physiological changes. For example, some female adolescents with autism are prone to epileptic seizures around the 14th day of their menstrual cycle, coinciding with a peak in luteinizing hormone (LH) levels [63]. This highlights a chain of events where disturbances in biological rhythms, compounded by heightened sensitivity to environmental stimuli, can increase arousal and physiological stress, ultimately triggering seizures in some children with autism [67].

Similarly, cortisol, a hormone that follows a diurnal cycle, is often dysregulated in ASD. While cortisol typically peaks in the morning and decreases throughout the day, individuals with ASD often display a blunted or abnormal cortisol rhythm, which may contribute to the hyperarousal and stress reactivity seen in these patients [68]. These hormonal disruptions suggest that circadian rhythm dysfunction may be a core feature of ASD, influencing not only sleep but also emotional regulation and social behaviour [20].

On a molecular level, ASD is associated with the dysregulation of several pathways involved in circadian rhythm regulation [69]. One of the most studied pathways is the canonical *WNT/β-catenin* pathway, which plays a crucial role in both neurodevelopment and the regulation of circadian rhythms [70,71,72].

Dysregulation of this pathway has been implicated in the metabolic reprogramming observed in ASD. Upregulation of *WNT/β-catenin* signalling has been linked to aberrant brain development, including altered neuronal connectivity and synaptic plasticity, both of which are specific features of autism [70,73]. Moreover, the *WNT/β-catenin* pathway interacts with circadian clock genes, suggesting that disruptions in this pathway may contribute to the circadian abnormalities seen in ASD [70,73].

*CLOCK* and *BMAL1* drive the expression of *PER* and *CRY* genes, essential components of the circadian feedback loop [74,75]. In ASD, mutations or dysregulation of these circadian genes have been observed: for example, the *PER1* gene, which is critical for the sleep–wake transition, has been found to be mutated in some individuals with ASD [76]. These mutations are thought to disrupt the normal oscillatory behaviour of circadian clocks, leading to abnormalities in sleep patterns, hormone secretion, and potentially other physiological rhythms [63].

Moreover, genetic studies have pointed to mutations in circadian regulators such as *NR1D1* (Rev-erbα), a gene that inhibits *BMAL1* expression and modulates circadian cycles [62]. Disruption of these pathways leads to the widespread circadian dysfunction seen in ASD, further emphasizing the importance of molecular chronobiology in understanding the disorder [77].

The clinical implications of circadian dysregulation in autism extend beyond sleep disturbances, influencing a range of behavioural symptoms [78]. Individuals with ASD often exhibit increased sensitivity to environmental stimuli, repetitive behaviours, and social withdrawal. These symptoms may be exacerbated by the disrupted synchronization of internal clocks with external environmental cues, such as the light–dark cycle [20].

One key manifestation of circadian disruption in ASD is sleep disturbance, which is reported in up to 80% of children with the condition [64]. Common sleep-related issues include delayed sleep onset, frequent nocturnal awakenings, reduced total sleep time, and early morning awakenings. Sleep disturbances not only worsen daytime behavioural issues such as irritability and hyperactivity but also interfere with cognitive development and learning, making them a critical area for intervention [65].

Moreover, sleep problems in ASD have been correlated with increased severity of repetitive behaviours and reduced social engagement [79]. For example, Goldman et al. (2011) assessed the relationship between sleep and behaviour in 1784 children aged from 2 to 18 years with a confirmed diagnosis of ASD [79]. Their findings indicated that poor sleepers exhibited a higher incidence of behavioural problems compared to good sleepers, with over three-quarters of the children reporting issues with attention and social interactions [79]. Internal desynchronization caused by circadian misalignment may aggravate repetitive motor behaviours and insistence on sameness, as individuals with ASD struggle to adapt to changes in their external or internal environments [68].

Given the profound impact of circadian dysfunction on ASD, chronotherapeutic interventions have emerged as a promising avenue for treatment.

### 4.2. Attention-Deficit/Hyperactivity Disorder

Attention-deficit/hyperactivity disorder (ADHD) is a complex neurodevelopmental disorder characterized by inattention, hyperactivity, and impulsivity [80,81]. However, beyond these core symptoms, recent research highlights the significant role that circadian rhythm disruptions play in the pathophysiology and symptomatology of ADHD [23,82,83].

The circadian system plays a crucial role in regulating the body’s daily physiological, behavioural, and cognitive processes [84].

ADHD is increasingly being recognized as a disorder with significant circadian dysregulation [85]. Several studies have shown that individuals with ADHD exhibit disturbances in their circadian rhythm, particularly in relation to their sleep–wake cycles [86]. One of the most common findings is a phase delay, where individuals experience a delay in their endogenous circadian markers, such as dim light melatonin onset (DLMO) [83,87]. In individuals with ADHD, the secretion of melatonin often occurs later in the evening, resulting in delayed sleep onset, reduced sleep duration, and difficulty waking in the morning [85,87].

The phase delay observed in ADHD aligns with a preference for eveningness, or “night owl” behaviour, a chronotype that is more prevalent in individuals with the disorder [83,88,89]. Studies using measures such as the Morningness–Eveningness Questionnaire (MEQ) and the Munich Chronotype Questionnaire (MCTQ) consistently show that ADHD patients tend to score higher on eveningness [87,90]. In the general population, eveningness is associated with altered emotionality, and ADHD-related traits such as apathetic, volatile, and disinhibited temperaments are linked to evening orientation, as is sensation-seeking behaviour [91]. This phase delay not only affects sleep patterns but also exacerbates core ADHD symptoms, such as inattention, hyperactivity, and impulsivity, particularly during the daytime when optimal cognitive function is required [88,89].

Light exposure is the primary external cue that synchronizes the circadian clock with the external environment, a process known as entrainment [86]. Light influences circadian rhythms through a pathway that involves intrinsically photosensitive retinal ganglion cells (ipRGCs), which detect changes in ambient light and relay this information to the SCN [92]. In individuals with ADHD, there may be alterations in light sensitivity or reduced exposure to natural light, which can disrupt the normal entrainment of circadian rhythms [83,85]. This misalignment between internal circadian rhythms and external environmental cues can lead to a range of behavioural and cognitive disruptions [88,89].

Interestingly, it has been suggested that individuals with ADHD may have a diminished sensitivity to morning light, which further contributes to delayed circadian phase [83]. This has significant implications for therapeutic interventions aimed at realigning the circadian clock in ADHD patients [23,86].

Several studies have identified polymorphisms in clock genes that are associated with both ADHD symptoms and circadian phase delays [85,93]. For instance, single nucleotide polymorphisms (SNPs) in the *CLOCK* and *PER3* genes have been linked to increased eveningness and delayed sleep onset in ADHD populations [87,88,89]. These genetic variants may alter the normal functioning of the molecular clock, leading to the chronobiological disturbances seen in ADHD [83].

Moreover, disruptions in the regulation of dopamine, that plays a key role in ADHD, have also been linked to circadian rhythm dysfunction [87,93,94]. Dopamine release is under circadian control, and alterations in dopaminergic pathways can affect both circadian timing and the expression of ADHD symptoms [83,88,89]. Circadian and dopaminergic systems are closely interconnected in the regulation of attention, impulsivity, and behaviour [86].

Chronotype, which refers to an individual’s preference for morning or evening activity, is strongly influenced by circadian rhythms [83,88,89]. In ADHD, the tendency toward a later chronotype has been associated with impaired cognitive performance during the morning hours [81]. This has important implications for both educational and occupational settings, where individuals with ADHD may struggle to perform at their best due to misaligned circadian timing [88,89]. Furthermore, subtype differences in the prevalence of sleep-onset insomnia have been indicated, with a decreased number of adults with the inattentive ADHD subtype displaying symptoms of sleep-onset insomnia compared to other subtypes [95]. Inattentive subtype patients not suffering from sleep-onset insomnia exhibited longer sleep duration and more stable sleep–wake rhythms compared to those with sleep-onset insomnia [96]. This aligns with previous reports that inattentive subtypes of ADHD are sleepier during the day and sleep for longer durations at a time, with dysregulation of the melatonin rhythm potentially mediating these associations [95,97].

Studies have shown that individuals with ADHD who have a later chronotype exhibit poorer performances in working memory, sustained attention, and executive function tasks, particularly in the early part of the day [83,85]. This misalignment between cognitive demand and circadian alertness contributes to the characteristic difficulties that are commonly seen in this population [88,89].

While much of the research on ADHD and circadian rhythms focuses on sleep, it is important to recognize that circadian disruption in ADHD affects a wide range of physiological processes beyond the sleep–wake cycle [81,92]. For example, Bijlenga et al. (2013) have recently postulated that the higher than expected prevalence of photophobia in ADHD may reflect a deficit in non-visual photic transmission associated with the circadian system, and that such a change could lead to the phase alterations observed in this conditions [98]. Circadian disturbance is further implicated in ADHD by findings that seasonal affective disorder (SAD), a form of depression intimately linked to circadian dysfunction [99], is significantly comorbid with this population [93,95,98,100]. For example, hormonal rhythms, including cortisol secretion, which follows a diurnal pattern, may be disrupted in ADHD [83,101]. 

There is evidence of blunted cortisol rhythms, which may contribute to daytime fatigue and cognitive difficulties [87]. Sex differences in the stress response have been identified in childhood ADHD, with elevated early morning cortisol levels in boys and decreased levels in girls [102].

Furthermore, circadian dysregulation in ADHD can affect metabolic processes, mood regulation, and cardiovascular function, all of which are under circadian control [81,92]. The pervasive nature of circadian dysfunction in ADHD underscores the importance of considering chronobiological factors in both the diagnosis and treatment of the disorder [83].

### 4.3. Post-Traumatic Stress Disorder

Post-traumatic stress disorder (PTSD) in children and adolescents is a significant mental health condition that arises after exposure to traumatic events [103]. This condition is marked by symptoms of re-experiencing, avoidance, hyperarousal, and mood alterations, all of which impair daily functioning [80]. Factors such as the type of trauma, the developmental timing of exposure, gender, pubertal development, social context, and psychopathology are hypothesized to contribute to the divergent findings in PTSD outcomes [104,105,106]. 

Emerging research highlights the role of circadian rhythm dysregulation, offering new insights into the chronobiological aspects of PTSD in younger populations [24]. Circadian rhythms are significantly affected in children with PTSD, often manifesting as delayed sleep phases and attenuated circadian amplitude [107]. Studies using actigraphy demonstrate that these children have more disrupted circadian rhythms compared to controls, with delayed sleep onset and irregular physical activity patterns during the day [108,109]. Children who experience early-onset trauma, particularly those who develop PTSD, often exhibit robust circadian rhythms with hyperactivity, while those without PTSD show attenuated rhythms [107]. Dysregulation of these rhythms may exacerbate PTSD symptoms and contribute to long-term psychiatric complications, such as depression and anxiety [110,111].

The hypothalamic–pituitary–adrenal (HPA) axis, responsible for regulating the body’s stress response, is deeply intertwined with circadian rhythms. In children with PTSD, the typical circadian pattern of cortisol secretion can be significantly disrupted [112]. Some studies have found elevated afternoon and evening cortisol levels in children with PTSD, indicating a flattened diurnal slope of cortisol secretion [113,114]. This flattening of cortisol rhythms has been linked to sustained hyperarousal and a heightened stress response, worsening PTSD symptoms such as sleep disturbances, mood dysregulation, and emotional resilience, together with difficulties with concentration and cognitive functioning [106,115,116,117,118].

The timing and type of trauma play a crucial role in the development of PTSD. Children exposed to chronic stress or multiple traumatic events are more likely to develop severe PTSD symptoms and show profound circadian rhythm disturbances [119]. For example, the timing of first trauma exposure has been associated with different diurnal secretion patterns in boys and girls [120,121].

The developmental stage at the time of trauma exposure also critically influences cortisol dysregulation. Younger children, especially those under 10, tend to have higher cortisol levels post-trauma compared to adolescents [122]. These elevated cortisol levels in younger children may be associated with hyperarousal and heightened stress reactivity, while adolescents tend to exhibit variable cortisol responses to trauma [122,123].

Cortisol profiles differ between children and adults with PTSD. In adults, studies often report low or normal baseline cortisol levels, whereas children frequently have elevated cortisol levels, especially in the evening [122,124]. Although cortisol levels in paediatric patients may normalize over time, noradrenaline concentrations often remain high, contributing to sustained hyperarousal [125,126].

Sleep disturbances are common and persistent in children and adolescents with PTSD, affecting more than 50% of patients after a traumatic event [109,127]. These disturbances include insomnia, nightmares, night terrors, fragmented sleep, and altered sleep architecture, particularly in rapid eye movement (REM) sleep [128]. Nightmares, in particular, are a hallmark of PTSD, affecting 50% to 80% of children after trauma [129,130].

Polysomnographic studies indicate that children with PTSD experience increased sleep fragmentation, elevated wake-after-sleep onset (WASO), and micro-arousals, all of which correlate with PTSD severity [109,131]. Additionally, environmental stressors exacerbate these disturbances, suggesting that interventions should include environmental considerations and psychoeducation for both children and their parents [131,132].

Gender plays a critical role in how PTSD manifests in relation to chronobiology and biorhythm. Studies consistently show that girls are more susceptible to trauma-related cortisol dysregulation and sleep disturbances than boys [133]. This heightened vulnerability is partly attributed to hormonal changes during puberty that interact with the HPA axis [134]. Girls with PTSD often exhibit elevated cortisol levels in response to trauma-related stimuli, linked to re-experiencing and hyperarousal symptoms [112]. Longitudinal increases in cortisol levels have been associated with higher anxiety in adolescent girls [135]. Similarly, Trickett et al. (2010) observed that girls who experienced trauma showed different developmental trajectories in cortisol regulation compared to boys, potentially influencing PTSD outcomes [136]. Furthermore, Luo et al. (2012) collected hair samples from adolescent girls exposed to a devastating earthquake in China [137]. Compared to controls, girls in the quake area had elevated cortisol levels for several months following the event. Subsequently, girls who developed PTSD diverged, exhibiting markedly lower hair cortisol levels compared to trauma-exposed girls without PTSD [137]. In contrast, boys may show lower morning cortisol levels, reflecting hypoactivation of the HPA axis [106]. These gender differences underscore the need for tailored, gender-specific approaches to PTSD treatment. Circadian rhythm dysregulation is a core feature of paediatric PTSD, contributing to the severity and persistence of the disorder. Altered cortisol patterns, delayed chronotypes, and long-term disruptions of the HPA axis highlight the importance of considering chronobiological factors in the diagnosis and treatment of PTSD in younger populations.

**Figure 1:**
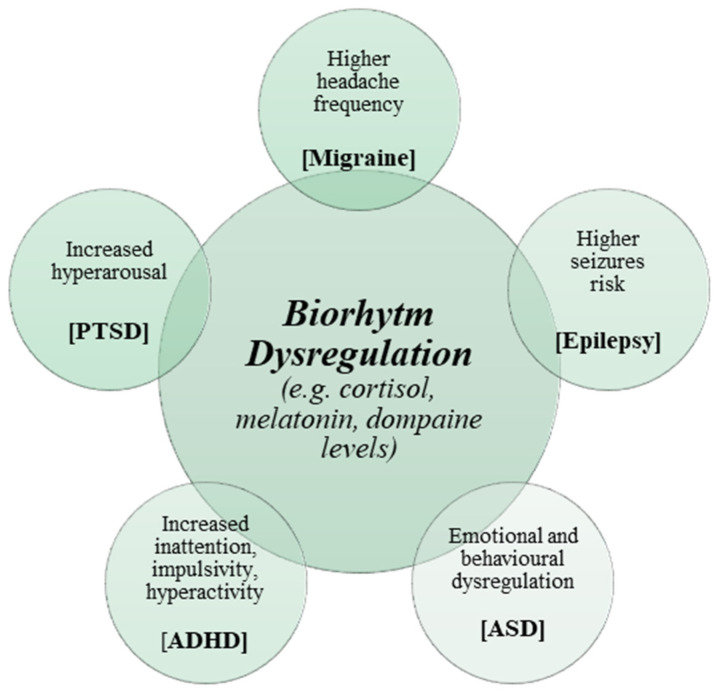
Potential consequences of biorhythm dysregulation in paediatric neurological and neuropsychiatric disorders. Legend: ASD: autism spectrum disorder; ADHD: attention-deficit/hyperactivity disorder; PTSD: Post-traumatic stress disorder.

Potential consequences of biorhythm dysregulation in paediatric neurological and neuropsychiatric disorders are shown in Figure 1.

## 5. Chronotherapy and Disorder-Specific Interventions

**Figure 2:**
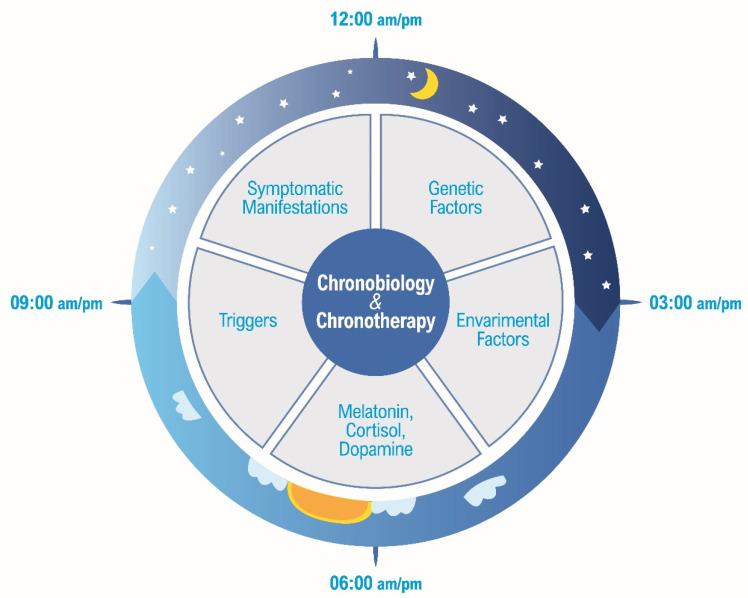
Interdependent chronobiological factors to consider in chronotherapy.

Chronotherapy, which involves synchronizing therapeutic interventions with the body’s circadian rhythms, has emerged as a promising approach for managing the aforementioned paediatric neurological conditions [101]. Interdependent chronobiological factors to consider in chronotherapy are shown in Figure 2. 

Central to these interventions are strategies that are applicable across these disorders, such as melatonin supplementation and the enhancement of sleep hygiene practices [65,87,138]. Melatonin, a key regulator of the sleep–wake cycle, has been extensively explored for its potential to improve sleep quality and regulate circadian rhythms in these populations [65,66,139,140]. Interventions focused on establishing consistent sleep routines, optimizing the sleep environment, and promoting regular exposure to natural light are fundamental components that can benefit children across these conditions [92,93,132,141,142].

For migraines in children, enhancing sleep hygiene has been shown to significantly reduce headache frequency [143]. Bellini et al. (2013) proposed a specialized sleep hygiene program targeting sleep depth, hypothesizing that excessive sleep depth could be responsible for migraine attacks [144]. Their technique, called “sleep rationing”, involves reducing total sleep time and relaxed sleep, effectively decreasing both REM sleep and slow-wave sleep [144]. This method has been successful in reducing both the intensity and severity of migraine attacks. Moreover, melatonin supplementation, particularly for those with low melatonin levels or circadian rhythm disruptions, offers promise in improving both sleep quality and migraine outcomes [38,139]. Although further research is necessary, these interventions represent a non-invasive approach to managing paediatric migraines.

In the context of epilepsy, chronotherapy aims to align antiepileptic drug administration with periods of greatest seizure susceptibility, potentially reducing seizure frequency [145]. Although results regarding melatonin’s efficacy in reducing seizures are mixed, its role in regulating circadian rhythms suggests potential benefits in improving sleep quality for paediatric epilepsy patients [146,147]. In a systematic review and meta-analysis of 2024, Liu et al. show that adjunctive treatment with melatonin improved sleep latency and seizure severity compared to placebo treatment [146]. Furthermore, addressing sleep disorders through interventions such as continuous positive airway pressure (CPAP) for obstructive sleep apnoea (OSA) or cognitive behavioural therapy (CBT) for insomnia could significantly enhance outcomes for these children [59].

In autism spectrum disorder (ASD), melatonin has demonstrated potential benefits beyond improving sleep, including enhancements in social behaviour and reductions in anxiety [63]. A systematic review and meta-analysis found that the use of melatonin in individuals with ASD was associated with significant improvements in sleep parameters, such as increased sleep duration and decreased sleep onset latency [65]. Additionally, melatonin appeared to improve daytime behaviour in some individuals with ASD, with minimal to no side effects [65]. Behavioural interventions that focus on synchronizing biological rhythms with external environmental cues, such as the Early Start Denver Model (ESDM), emphasize the importance of synchrony in motor, emotional, and social rhythms [148]. This approach aims to align the child’s internal rhythms with those of caregivers and the broader environment, promoting better adaptation and reducing stress [148]. Emerging research also suggests targeting molecular pathways involved in circadian regulation, such as the *WNT/β-catenin* pathway, as a therapeutic strategy to restore normal circadian function and alleviate symptoms [70,73].

In ADHD, the strong association between circadian disruption and symptomatology makes chronotherapy a promising treatment avenue [85,87]. Bright light therapy (BLT), administered in the morning, can effectively shift circadian rhythms earlier, realign the circadian clock, promote earlier sleep onset, and improve daytime alertness [86,149]. BLT is a treatment that involves exposure to high-intensity artificial light to regulate circadian rhythms and alleviate symptoms of mood and sleep disorders [141,150]. By simulating natural sunlight, BLT helps reset the body’s internal clock, making it effective for conditions like seasonal affective disorder (SAD) and certain circadian rhythm sleep disorders like delayed sleep phase disorder, commonly observed in ADHD patients [151]. Supporting this, an open-label trial in adult population by Rybak et al. (2006) demonstrated that morning BLT was associated with significant improvements in both subjective and objective measures of baseline ADHD pathology, as well as improvements in mood symptoms and a significant phase advance in circadian rhythms [93]. This suggests that a shift toward an earlier circadian phase with BLT could be a predictor of improvement in both subjective and objective ADHD measures. Melatonin supplementation in the early evening can advance the circadian phase, leading to earlier sleep onset and improved sleep quality [96]. However, the timing and dosage of melatonin are critical, as improper use can exacerbate circadian misalignment [87,152]. Behavioural strategies that promote consistent sleep–wake schedules and increased exposure to natural light during the day may further mitigate circadian disturbances [149,151].

For paediatric PTSD, chronotherapeutic interventions such as BLT can shift the circadian phase earlier, aligning biological and social clocks, which may alleviate symptoms [118,142,153]. If sleep is one of the pathways through which the long-term effects of trauma are perpetuated, interventions aimed at restoring greater normalcy in sleep–wake cycles may be useful in alleviating PTSD symptoms [128]. Charuvastra (2009) demonstrated that, in children with PTSD, sleep may represent a potential behavioural target for intervention to reduce the long-term negative health effects of childhood trauma [154]. Melatonin supplementation may also help regulate sleep–wake cycles, though careful management of timing and dosage is essential [140,155]. These interventions target the circadian system to improve both sleep and mood regulation, potentially enhancing PTSD outcomes.

A synthesis of chronobiological factors and therapeutic prospects in the aforementioned paediatric neurological and neuropsychiatric disorders is reported in Table 1.

## 6. Discussion

This narrative review has highlighted the profound influence of chronobiology on the symptomatology of various paediatric neurological and neuropsychiatric conditions, including migraine, epilepsy, autism spectrum disorder (ASD), attention-deficit/hyperactivity disorder (ADHD), and post-traumatic stress disorder (PTSD). Despite the distinct clinical presentations of these disorders, a common thread emerges: disruptions in circadian rhythms and biological clocks play a pivotal role in their pathophysiology and symptom expression.

The evidence gathered, including dysregulation of melatonin and cortisol secretion, alterations in core clock genes (e.g., *CLOCK*, *BMAL1*, *PER*, *CRY*) [52,53,74,75,87,88], and disturbances in neurotransmitter systems [34,63,87,116,117,118], provides compelling clues that highlight the pivotal role of the circadian system in neurodevelopmental and neuropsychiatric processes. These disruptions contribute to a spectrum of symptoms, from sleep disturbances and cognitive impairments to mood dysregulation and heightened stress responses.

Understanding these common biological underpinnings offers a unique opportunity to transform therapeutic strategies. Chronotherapy, which synchronizes treatment interventions with the body’s natural rhythms, has shown promise across multiple conditions. In a 2021 review, Lee et al. summarized recent advances in chronobiology, with a particular focus on disease models that could potentially benefit from circadian rhythm-based therapies [156]. The authors discussed both non-pharmacological and pharmacological interventions targeting circadian clocks in disease contexts, including Chrono-Phototherapy, Chrono-Diet, Chrono-Exercise, and the use of circadian components as drug targets [156]. Transversal therapies such as melatonin supplementation and enhancements in sleep hygiene have demonstrated efficacy in improving sleep quality and reducing symptom severity. Disorder-specific interventions, including BLT for ADHD [86,149] and behavioural synchrony approaches like the ESDM in ASD [148], further highlight the potential of tailored chronotherapeutic strategies.

However, while the potential of chronotherapy is clear, several limitations must be considered. One critical aspect is the side effects associated with chronotherapeutic interventions. For instance, while melatonin supplementation can improve sleep, its prolonged use in children could potentially affect pubertal development or cause daytime sleepiness [157]. Likewise, the use of bright light therapy may lead to adverse effects like eye strain or even exacerbation of mood symptoms in some individuals [141]. It is important that the clinical implementation of these therapies include careful monitoring and individualized adjustments.

The integration of chronobiological principles into clinical practice necessitates a paradigm shift in the approach to these paediatric conditions. By acknowledging and targeting the circadian disruptions inherent in these disorders, clinicians can move beyond symptom management toward interventions that address fundamental physiological processes. This holistic perspective not only holds the potential for treatments that are more effective but also for improving the overall quality of life for affected children and their families.

Moreover, the application of chronotherapy extends beyond clinical efficacy. Molecular insights into how circadian rhythms regulate the expression of genes involved in neurotransmission, neuroplasticity, and immune responses are integral to further understanding its therapeutic mechanisms. Studies investigating how circadian rhythms influence specific brain areas, such as the prefrontal cortex in ADHD [96] or the amygdala in PTSD [158], could provide a more detailed understanding of how timing interventions can influence brain function and behaviour. The interplay between circadian genes and neuroplasticity, for instance, may offer novel therapeutic targets for more effective treatments [159].

These scientific insights have direct implications for clinical practice. Administering medications at times that align with patients’ circadian rhythms can enhance efficacy and minimize side effects. For example, prescribing stimulant medications for ADHD earlier in the day may optimize attention during peak cognitive periods while reducing sleep disturbances at night. Similarly, scheduling physiotherapy, neuropsychomotor therapy, or psychotherapy sessions when patients are most alert and receptive can maximize therapeutic benefits.

Despite the promise of chronotherapy, its adoption in routine clinical settings faces significant barriers. A major obstacle is the accessibility of therapies, such as specialized light devices or personalized chronotherapy regimens [160]. Furthermore, ensuring patient compliance, especially in paediatric populations, can be challenging. Specifically, adhering to strict timing of medications or behavioural interventions may be difficult for both patients and their families. It is therefore crucial that future research explore strategies to improve engagement with chronotherapy protocols and develop guidelines that make these therapies more accessible to diverse populations. These concrete applications of chronotherapeutic principles could enable more personalized and effective treatment plans tailored to individuals’ biological rhythms [161].

Advancing the field of chronotherapy requires concerted efforts to address its inherent challenges. Future research must encompass diverse populations, including varying socio-economic statuses, geographic regions, and genetic backgrounds, to identify subgroups most likely to benefit from circadian-based interventions [162]. Elucidating the molecular mechanisms by which circadian rhythms influence neural substrates involved in cognition, mood regulation, and stress response is paramount; such insights could facilitate the development of more precisely targeted treatments [163]. Personalization of therapeutic protocols, tailored to individual genetic profiles, lifestyles, and environmental factors, holds promise for optimizing outcomes through alignment of medication schedules with patients’ intrinsic biological rhythms [164].

Effective clinical implementation hinges on overcoming practical obstacles related to accessibility, affordability, and patient adherence. Developing user-friendly technologies and mobile health solutions may simplify the application of chronotherapy and enhance compliance. Moreover, long-term, large-scale longitudinal studies are necessary to evaluate the sustained efficacy of chronotherapeutic interventions on symptom amelioration and quality of life across different age groups and neuropsychiatric disorders [165].

In summary, while the integration of chronobiological principles into clinical practice presents profound opportunities for more personalized and efficacious treatments, it is imperative to meticulously consider challenges pertaining to potential side effects, accessibility, and adherence. Deeper molecular research is indispensable to fully comprehend how circadian rhythms modulate brain function and behaviour. By elucidating these interactions, we can empower existing therapies and foster the development of novel interventions that may drastically alter the course of neuropsychiatric pathologies. As these avenues are explored, we aspire not only to innovate therapeutic strategies but also to enrich our understanding of circadian biology’s role in neurological and neuropsychiatric health throughout the lifespan.

## 7. Conclusions

This narrative clinical review underscores the critical role of chronobiology in shaping symptom expression and therapeutic potential for paediatric neurological and neuropsychiatric disorders. Recognizing circadian disruptions as central to the pathophysiology of conditions like migraine, epilepsy, ASD, ADHD, and PTSD allows for a paradigm shift from symptom management to interventions targeting foundational biological rhythms. Future research should focus on elucidating the intricate relationships between circadian biology and neurological function. Unravelling the molecular pathways involved and identifying individual differences in circadian timing and genetic backgrounds are essential for developing targeted chronotherapeutic interventions. Emphasizing personalized medicine, chronotherapy allows treatments to be tailored to each individual’s unique circadian profile, maximizing therapeutic efficacy while minimizing side effects.

Ultimately, embracing the chronobiological dimensions of these conditions holds the potential to significantly enhance therapeutic outcomes and foster a more unified and effective approach to paediatric neurological and neuropsychiatric care. By integrating chronotherapy into clinical practice, we may not only optimize existing treatments but also facilitate the development of novel interventions that could profoundly alter the course of these disorders.

This personalized approach could inaugurate a new era in paediatric medicine, where interventions are synchronized with the biological rhythms of each patient, thereby improving quality of life and long-term health outcomes. 

## 8. Limitations

While this narrative review provides a comprehensive synthesis of existing research on the influence of circadian rhythms on paediatric neurological and neuropsychiatric conditions, several limitations should be acknowledged. First, we did not perform a formal qualitative appraisal of the included studies. As a result, the varying methodological quality and potential biases inherent in individual studies were not systematically evaluated. This may affect the overall strength of the conclusions drawn. Future research could benefit from incorporating standardized critical appraisal tools to assess study quality, thereby enhancing the reliability of synthesized findings.

Second, our review includes a diverse range of study types. While this approach allowed us to capture a broad spectrum of existing knowledge, it also introduces variability in the level of evidence presented. Many included studies have limitations like small sample sizes, lack of control groups, or potential confounding factors that may influence results. These factors could limit the generalizability of our findings and should be considered when interpreting the implications of this review.

Moreover, given the wide and evolving nature of this topic, some areas lack extensive research, resulting in gaps in the literature. The field of paediatric chronobiology is still developing, and high-quality, large-scale studies are needed to confirm and expand upon the insights discussed herein.

Despite these limitations, our review highlights important patterns and potential avenues for integrating chronobiological principles into clinical practice. By synthesizing existing research, we aim to stimulate further studies and encourage the consideration of chronotherapy-based interventions for paediatric neurological and neuropsychiatric conditions.

## Figures and Tables

**Table 1:** Chronobiological factors in paediatric neurological and neuropsychiatric disorders.

Disorder	Circadian Rhythmicity Observations	Neurobiological Implications	Symptoms/ Behavioural Manifestations	Chronotherapeutic Considerations	Timing of Interventions
**Migraine**	Higher occurrence in early morning and late afternoon; melatonin reduction in the evening.	Hypothalamic dysfunction linked with SCN disruption; serotonin and dopamine dysregulation.	Sleep disturbances (e.g., insomnia, frequent night awakenings); evening chronotype linked to higher headache frequency.	Melatonin supplementation; sleep hygiene; structured light exposure.	Melatonin: evening; Light exposure: morning/early afternoon.
**Epilepsy**	Seizures more likely in early morning or at night due to increased neuronal excitability from *BMAL1* and *CLOCK* activation.	Developing brain’s sensitivity to circadian misalignments; seizure-type-specific peaks.	Sleep issues (e.g., insomnia, daytime sleepiness); seizure-induced awakenings; periodic cortical excitability fluctuations.	Scheduled antiepileptic drug administration; CBT for sleep issues.	Antiepileptic drugs: aligned with circadian vulnerability phases.
**ASD**	Evening melatonin reduction; irregular cortisol cycle with high afternoon levels.	Disruptions in *CLOCK*, *BMAL1*, *PER*, and *CRY* genes affecting neurodevelopment.	Emotional dysregulation, anxiety, social withdrawal, repetitive behaviours, and hyperarousal worsened by circadian disruption.	Melatonin supplementation; behavioural synchronization therapies (e.g., Early Start Denver Model).	Melatonin: evening; synchronization therapies: aligned with daily routines.
**ADHD**	Delayed melatonin peak in late afternoon; “eveningness” chronotype reduces sunlight exposure.	High evening dopamine, *CLOCK*, and *PER3* activity delay sleep onset; dopamine dysregulation impacts attention and impulse control.	Inattention, impulsivity, hyperactivity, sleep-onset insomnia, and daytime fatigue.	Morning bright light therapy; melatonin; strict sleep-wake routines; scheduling activities with circadian rhythms.	Bright light: morning; melatonin: evening.
**PTSD**	Hyperactive HPA axis with elevated cortisol and noradrenaline levels in afternoon/evening.	HPA axis and circadian disruptions contribute to hyperarousal.	Insomnia, nightmares, mood dysregulation, heightened stress sensitivity and hyperarousal.	Chronotherapy (morning light exposure); melatonin; environmental adjustments for optimal sleep.	Light exposure: morning; melatonin: evening.

Legend: ASD: autism spectrum disorder; ADHD: attention-deficit/hyperactivity disorder; PTSD: post-traumatic stress disorder; CBT: cognitive behavioural therapy; HPA: hypothalamic–pituitary–adrenal.

## Data Availability

Data will be available on request to the corresponding author.
